# Volume XIII. Our Thirteenth Voyage

**Published:** 1897-07

**Authors:** 


### VOLUME XIII. OUR THIRTEENTH VOYAGE.

The TEXAS MEDICAL JOURNAL salutes its scores of readers all over the land; sends greeting, and thanking them for their zealous support and aid in making the *Red Back* "the best medical journal in the South," announces with pleasure that with our *June* number we completed our twelfth year of success. With this issue we begin the ceaseless round, and encouraged by a liberal patronage, and cheered by a most enthusiastic reception wherever the JOURNAL's brilliant shining face is seen, we address ourself to the task anew which the JOURNAL set out to accomplish, dedicate anew to the cause of organization our best energies and endeavors. Congratulating ourselves and our constituency on what has been accomplished—and it has been much—we are encouraged to hope for further success. When the JOURNAL stepped into the ring twelve years ago to make war on every species of quackery, and to advocate a unity of forces, the organization of the rank and file of the medical corps, there were not three local medical organizations intact. Now there is a flourishing enthusiastic society in every district, and still the good work goes bravely on. The JOURNAL has been universally pronounced a credit to Texas. It has become known far and near as the champion of legitimate medicine and higher education, whose sole mission is the elevation and advancement, by means of organization, of the regular profession, for the extinction of quackery, and it has become a terror to evil doers. In organization lies our only hope of exerting sufficient influence to secure such medical legislation as is essential to put a stop to the abuse of the practice of medicine; to protect the people from the ravages of the vultures in human shape who, taking advantage of the absence of restraint in Texas, are preying upon the misfortunes and the credulity of the people. The reason of the repeated failures at medical legislation is found here. Once the State Association could assemble four or five hundred members—a mere tithe then—but now it is difficult to get together one hundred. We must renew our efforts, and cease not until every reputable doctor in the State is enrolled in a grand State Association, and then their voice will be heeded. Let every reader consider himself especially appointed to work in the cause of organization and begin at home. Let there be a society in every county, and let every society be represented by delegate in a State Association, and we may then hope to make the influence of the profession felt; then we may hope to get State recognition—something the Association has never had—and no longer be regarded by the average legislator as belonging to a "school" of medicine. Let them know that there is but *one school* of medicine—the ancient and honorable calling of rational medicine; all others are mere pathies.

We send greeting to our brethren everywhere, and solicit their support and co-operation in the laudable work in which the JOURNAL is engaged.

